# Editorial: Optimizing opioid prescriptions in the emergency department

**DOI:** 10.1186/s12873-023-00880-0

**Published:** 2023-09-19

**Authors:** Ashraf A. Dahaba, Rishi S. Nannan Panday

**Affiliations:** 1 Department of Anaesthesiology and Intensive Care Medicine, Suez Canal University, Ismailia, 41522 Egypt; 2 Department of Internal Medicine, Section Acute Medicine, Amsterdam University Medical Centers, Amsterdam, Netherlands

**Keywords:** Opioids, Emergency Department, Pain management, Addiction, Nonsteroidal anti-inflammatory drugs NSAIDs

## Abstract

Optimizing opioid prescriptions in the emergency department is essential to address the opioid pandemic while ensuring patient wellbeing. This requires a comprehensive approach that includes exploring alternatives to opioids for pain management, identifying individuals at risk for opioid addiction, implementing evidence-based guidelines, and involving doctors in the management of opioid addiction.

The opioid pandemic has become a major public health crisis, leading to a significant increase in morbidity and mortality associated with the use of opioid pain relievers (OPRs). Efforts to address this crisis have primarily focused on reducing nonmedical OPR use, but the need for preventing and treating opioid addiction has often been overlooked [1]. Recently Purdue Pharma, owned by the renowned Sackler family, pleaded guilty to criminal charges and agreed to pay $8bn in opioid settlement to resolve a probe for their role in fueling America’s opioid crisis [2]. Overprescribing OPRs as a result of such practices has contributed to a sharp rise in opioid addiction, leading to an increase in overdose deaths and heroin use [1]. To effectively reduce opioid-related morbidity and mortality, a multifaceted public health approach is required, utilizing primary, secondary, and tertiary prevention strategies.

In the emergency department, severe pain is a common presenting complaint, and opioids have traditionally been the mainstay of treatment. However, it is crucial to explore alternatives to opioids for the treatment of severe pain in this setting. Non-opioid analgesics, such as nonsteroidal anti-inflammatory drugs (NSAIDs), acetaminophen, and regional anesthesia techniques, can be effective in managing pain without the risk of opioid addiction and associated adverse effects [3]. Implementing these alternatives can help reduce the reliance on opioids in the emergency department and minimize the potential for long-term opioid use, misuse, overdose, and death.

Certain factors make individuals more prone to opioid addiction. These include a history of substance abuse, mental health disorders, genetic predisposition, social and environmental factors. Understanding these risk factors is crucial for identifying individuals who may be at higher risk for opioid addiction and tailoring interventions accordingly [4]. It is essential to address the evolving trends in substance use, health, and social functioning to ensure the effectiveness of addiction treatment programs.

Optimizing opioid prescriptions in the emergency department requires a delicate balance between providing adequate pain relief and minimizing the risk of opioid-related harm. Implementing evidence-based guidelines and protocols can help guide clinicians in making appropriate prescribing decisions. These guidelines should emphasize the use of non-opioid analgesics as first-line treatment for pain whenever possible and promote judicious use of opioids for severe pain that is unresponsive to other interventions [5]. Additionally, implementing prescription drug monitoring programs can help identify patients who may be at risk for opioid misuse or diversion [3].

Doctors play a crucial role in the management of opioid addiction. They have the opportunity to identify and intervene early in cases of opioid misuse or addiction, particularly in the emergency department setting. Initiating treatment and providing referrals to addiction specialists and resources can help individuals with opioid addiction access the care they need [6]. Furthermore, doctors can contribute to the prevention of opioid addiction by promoting non-opioid alternatives for pain management, and educating patients about the risks and benefits of opioid use [7].

In conclusion, optimizing opioid prescriptions in the emergency department is essential to address the opioid pandemic while ensuring patient wellbeing. This requires a comprehensive approach that includes exploring alternatives to opioids for pain management, identifying individuals at risk for opioid addiction, implementing evidence-based guidelines, and involving doctors in the management of opioid addiction. In this special issue we will explore these different subjects.

## Data Availability

Not applicable.

## Abbreviations

OPRs: opioid pain relievers
NSAIDs: nonsteroidal anti-inflammatory drugs
